# The association between body mass index and recovery from post-traumatic stress disorder after the nuclear accident in Fukushima

**DOI:** 10.1038/s41598-021-84644-5

**Published:** 2021-03-05

**Authors:** Masato Nagai, Tetsuya Ohira, Masaharu Maeda, Seiji Yasumura, Itaru Miura, Shuntaro Itagaki, Mayumi Harigane, Kanae Takase, Hirooki Yabe, Akira Sakai, Kenji Kamiya

**Affiliations:** 1 Department of International and Community Oral Health, Tohoku University Graduate School of Dentistry, 4-1, Seiryo-machi, Aoba-ku, Sendai, Miyagi 980-8575 Japan; 2 Department of Epidemiology, Fukushima Medical University School of Medicine, Fukushima, Japan; 3 Radiation Medical Science Center for the Fukushima Health Management Survey, Fukushima Medical University, Fukushima, Japan; 4 Department of Disaster Psychiatry, Fukushima Medical University School of Medicine, Fukushima, Japan; 5 Department of Public Health, Fukushima Medical University School of Medicine, Fukushima, Japan; 6 Department of Neuropsychiatry, Fukushima Medical University School of Medicine, Fukushima, Japan; 7 Department of Public Health and Home Care Nursing, Fukushima Medical University, Fukushima, Japan; 8 Department of Radiation Life Science, Fukushima Medical University School of Medicine, Fukushima, Japan; 9 Hiroshima University, Higashihiroshima, Japan

**Keywords:** Psychology, Risk factors

## Abstract

Post-traumatic stress disorder (PTSD) and obesity share common risk factors; however, the effect of obesity on recovery from PTSD has not been assessed. We examined the association between body mass index (BMI) and recovery from PTSD after the Great East Japan Earthquake. We analyzed 4356 men and women with probable PTSD aged ≥ 16 years who were living in evacuation zones owing to the radiation accident in Fukushima, Japan. Recovery from probable PTSD was defined as Post-traumatic Stress Disorder Checklist-specific scores < 44. Using Poisson regression with robust error variance adjusted for confounders, we compared the prevalence ratios (PRs) and 95% confidence intervals (CIs) for this outcome in 2013 and 2014. Compared with point estimates for normal weight (BMI: 18.5–24.9 kg/m^2^), especially in 2013, those for underweight (BMI: < 18.5 kg/m^2^) and obesity (BMI: ≥ 30.0 kg/m^2^) tended to slightly increase and decrease, respectively, for recovery from probable PTSD. The multivariate-adjusted PRs (95% CIs) for underweight and obesity were 1.08 (0.88–1.33) and 0.85 (0.68–1.06), respectively, in 2013 and 1.02 (0.82–1.26) and 0.87 (0.69–1.09), respectively, in 2014. The results of the present study showed that obesity may be a useful predictor for probable PTSD recovery. Obese victims with PTSD would require more intensive support and careful follow-up for recovery.

## Introduction

On March 11, 2011, the Great East Japan earthquake (GEJE) caused a tsunami, as well as a radiation accident at the Fukushima Daiichi Nuclear Power Plant in Fukushima Prefecture, on the Pacific coast of northern Japan. Several studies have conducted to observe the effects of this complex disaster on the health of its victims. For example, the prevalence of overweight and obesity in this population, defined as body mass index (BMI) ≥ 25 kg/m^2^, increased dramatically after the disaster^1^, from 32.8% and 30.5% to 42.6% and 35.9% in evacuated men and women, respectively. Previous studies also reported a high prevalence of posttraumatic stress disorder (PTSD) symptoms (men: 18.6%, women: 24.9%), defined as Post-traumatic Stress Disorder Checklist-specific (PCL-S) scores ≥ 44 in 2012, almost one year after the disaster^2–4^.

Obesity and PTSD share several common risk and correlated factors such as diet^5–8^, biochemical values^8–12^, and personality^13–17^. Individuals with PTSD are likely to be obese and have an increased risk of weight gain^5,11,18^. However, to our knowledge, the effect of obesity on recovery from PTSD has not been reported. In this context, BMI may be a predictor for recovery from PTSD.

Therefore, this study examined the association between BMI and recovery from PTSD by longitudinal data from evacuees who lived in the evacuation zone owing to the nuclear accidents in Fukushima in 2011.

## Results

### Baseline characteristics by BMI

Table 1 shows baseline characteristics of the study participants according to BMI. The prevalence of PCL-S scores < 44 lowered with increased BMI in both 2013 and 2014. In underweight, the prevalence of women, younger participants, never smoked and current smoker, don’t drink or only rarely drinker, almost never having exercise habit, very dissatisfied about sleep or haven’t slept at all, independent about activities of daily living, and no damage in lived house were the highest compare with the other participants. Meanwhile, the mean PCL-S scores in 2012 and the prevalence of a history of cancer or cardiovascular disease, lose someone close in disaster, experience about tsunami, and experience about nuclear reactor accident (heard the explosion) were lowest in underweight.

**Table 1:** Baseline characteristics by BMI^†^ in 2012, Fukushima, Japan.

	BMI (kg/m^2^)			
	< 18.5	18.5 to < 25.0	25.0 to < 30.0	≥ 30.0
No. of participants	218	2614	1289	235
PCL-S^†^ scores < 44 in 2013 (%)	47.3	41.4	40.5	35.3
Missing	20.2	17.9	17.5	17.9
PCL-S scores < 44 in 2014 (%)	42.7	41.2	38.4	35.3
Missing	29.8	26.9	29.4	29.4
Men (%)	17.0	33.3	48.0	37.5
**Age (%)**				
< 30 years	17.0	5.5	2.4	7.7
30–39 years	17.0	11.6	6.8	11.9
40–49 years	16.5	9.4	7.3	16.6
50–59 years	11.9	16.3	16.5	18.3
60–69 years	22.5	28.7	31.7	27.7
≥ 70 years	15.1	28.5	35.3	17.9
Mean PCL-S scores in 2012 (SD^†^)	54.3 (8.9)	55.4 (9.6)	55.9 (9.9)	55.6 (10.0)
**Smoking (%)**				
Never smoked	62.8	61.7	57.5	57.5
Quit	11.9	20.2	25.6	20.4
Current smoker	23.9	15.3	13.1	19.2
Missing	1.4	2.9	3.8	3.0
**Drinking (%)**				
Don't drink or only rarely	61.0	51.0	48.3	56.2
Quit	3.7	3.7	3.7	3.8
Drink at least once a month	33.5	43.5	46.2	37.0
Missing	1.8	1.8	1.9	3.0
**Exercise habit (%)**				
Almost every day	12.4	16.9	15.7	8.1
2–4 times/week	17.4	24.2	27.6	21.3
Once/week	11.5	12.9	15.4	17.9
Almost never	57.8	43.8	39.2	51.5
Missing	0.9	2.2	2.2	1.3
**Sleeping habit (%)**				
Satisfied	8.7	10.0	12.4	6.4
Slightly dissatisfied	37.2	35.1	34.2	36.6
Quite dissatisfied	26.6	28.2	24.8	27.7
Very dissatisfied or haven't slept at all	13.3	9.1	10.8	12.8
Missing	14.2	17.6	17.8	16.6
**Education level (%)**				
Elementary school ・ Junior high school	15.1	23.8	32.5	20.4
High school	51.4	50.4	45.9	51.1
Vocational college/Junior college	26.2	16.3	13.3	17.0
University ・ Graduate school	6.0	5.7	4.5	6.8
Missing	1.4	3.7	3.8	4.7
Activities of daily living (%)				
Independent	97.3	96.6	95.4	96.2
Dependent	2.3	2.0	3.0	3.8
Missing	0.5	1.4	1.6	0.0
History of cancer or cardiovascular disease (%)	10.1	19.1	21.6	20.4
Missing	2.3	3.1	4.0	2.1
**House damage (%)**				
No damage	21.6	15.7	13.9	18.7
Partial damage or partial collapse	57.3	65.4	66.9	60.4
Partial but extensive collapse or total collapse	11.9	12.5	12.7	12.8
Missing	9.2	6.5	6.6	8.1
Lose someone close in disaster (%)	24.8	30.2	30.6	31.1
Missing	0.5	1.9	2.4	2.6
Experience about tsunami following the GEJE^†^ (%)	25.2	28.6	30.3	35.3
Experience about nuclear reactor accident (heard the explosion) following the GEJE (%)	65.1	69.9	69.7	71.9

^†^BMI, body mass index; PCL-S, Post-traumatic Stress Disorder Checklist-specific, SD; standard deviation, GEJE; the Great East Japan Earthquake.

### BMI and recovery from probable PTSD

Table 2 shows PRs with 95% CIs between BMI and recovery from probable PTSD. Compared with the point estimates for normal weight, those for underweight and obesity tended to slightly increase and decrease, respectively, for probable PTSD, especially in 2013. The multivariate-adjusted PRs (95% CIs) in the underweight and obesity were 1.08 (0.88–1.33) and 0.85 (0.68–1.06), respectively, in 2013 (p for trend = 0.367) and 1.02 (0.82–1.26) and 0.87 (0.69–1.09), respectively, in 2014 (p for trend = 0.524).

**Table 2:** PRs^†^ and 95% CIs^†^ of recovery from probable PTSD^†^ according to BMI^†^ in 2012.

	BMI (kg/m^2^)				P for trend^§^
	< 18.5	18.5 to < 25.0	25.0 to < 30.0	≥ 30.0	
**Year 2013**					
No. of participants	174	2147	1063	193	
No. of events (PCL-S^†^ scores < 44)	103	1083	522	83	
Crude PRs (95% CIs)	1.17 (1.03–1.34)	Reference	0.97 (0.90–1.05)	0.85 (0.72–1.01)	0.022
Sex-age-adjusted PRs (95% CIs)	1.11 (0.97–1.27)	Reference	1.00 (0.92–1.07)	0.83 (0.70–0.98)	0.104
Multivariate-adjusted PRs^‡^ (95% CIs)	1.08 (0.88–1.33)	Reference	1.00 (0.90–1.11)	0.85 (0.68–1.06)	0.367
**Year 2014**					
No. of participants	153	1910	910	166	
No. of events (PCL-S scores < 44)	93	1078	495	83	
Crude PRs (95% CIs)	1.08 (0.94–1.23)	Reference	0.96 (0.90–1.04)	0.89 (0.76–1.04)	0.083
Sex-age-adjusted PRs (95% CIs)	1.02 (0.89–1.16)	Reference	0.99 (0.92–1.06)	0.87 (0.74–1.02)	0.335
Multivariate-adjusted PRs (95% CIs)	1.02 (0.82–1.26)	Reference	0.99 (0.89–1.10)	0.87 (0.69–1.09)	0.524

^†^PR, prevalence ratio; CI, confidence interval; PTSD, post-traumatic stress disorder; BMI, body mass index; PCL-S, Post-traumatic Stress Disorder Checklist-specific.

^‡^Multivariate-adjusted PRs were adjusted for sex, age (< 30 years, 30–39 years, 40–49 years, 50–59 years, 60–69 years, or ≥ 70 years), PCL-S scores in 2012 (continuous), smoking (never smoked, quit, or current smoker), drinking (don't drink or only rarely, quit, or drink at least once a month), exercise habit (almost every day 2–4 times/week, once/week, or almost never), sleep habit (satisfied, slightly dissatisfied, quite dissatisfied, or very dissatisfied or haven't slept at all), education level (elementary school・junior high school, high school, vocational college/junior college, or university・graduate school), activities of daily living (independent or dependent), history of cancer or cardiovascular disease (yes or no), house damege (no damage, partial damage or partial collapse, or partial but extensive collapse or total collapse), lose someone close in disaster (yes or no), experience about tsunami following the GEJE (yes or no), experience about nuclear reactor accident (heard the explosion) following the GEJE (yes or no).

^§^P for trend was calculated by continuous valuable.

Table 3 shows the PRs and 95% CIs between BMI and recovery from probable PTSD in 2014 among participants who had probable PTSD until 2013. No trend in these associations was observed (p for trend = 0.889).

**Table 3:** PRs^†^ and 95% CIs^†^ of recovery from probable PTSD^†^ according to BMI^†^ in 2012 and 2013.

	BMI (kg/m^2^)				P for trend^§^
	< 18.5	18.5 to < 25.0	25.0 to < 30.0	≥ 30.0	
**Year 2014**					
No. of participants	42	741	355	70	
No. of events (PCL-S^†^ scores < 44)	15	257	117	27	
Crude PRs (95% CIs)	1.03 (0.68–1.56)	reference	0.95 (0.80–1.14)	1.11 (0.81–1.52)	0.906
Sex-age-adjusted PRs (95% CIs)	0.96 (0.64–1.45)	reference	0.98 (0.82–1.18)	1.08 (0.79–1.46)	0.839
Multivariate-adjusted PRs^‡^ (95% CIs)	0.99 (0.58–1.69)	reference	0.95 (0.76–1.19)	1.07 (0.71–1.60)	0.889

^†^PR, prevalence ratio; CI, confidence interval; PTSD, post-traumatic stress disorder; BMI, body mass index; PCL-S, Post-traumatic Stress Disorder Checklist-specific.

^‡^Multivariate-adjusted PRs were adjusted for sex, age (< 30 years, 30–39 years, 40–49 years, 50–59 years, 60–69 years, or ≥ 70 years), PCL-S scores in 2012 (continuous), smoking (never smoked, quit, or current smoker), drinking (don't drink or only rarely, quit, or drink at least once a month), exercise habit (almost every day 2–4 times/week, once/week, or almost never), sleep habit (satisfied, slightly dissatisfied, quite dissatisfied, or very dissatisfied or haven't slept at all), education level (elementary school・junior high school, high school, vocational college/junior college, or university・graduate school), activities of daily living (independent or dependent), history of cancer or cardiovascular disease (yes or no), house damege (no damage, partial damage or partial collapse, or partial but extensive collapse or total collapse), lose someone close in disaster (yes or no), experience about tsunami following the GEJE (yes or no), experience about nuclear reactor accident (heard the explosion) following the GEJE (yes or no).

^§^P for trend was calculated by continuous valuable.

These associations remained consistent after excluding participants aged < 20 years (Supplemental Table 1 and 2).

## Discussion

This study showed that BMI, especially obesity, predicts probable PTSD recovery in one year later. Meanwhile, the BMI in the participants who did not recover from probable PTSD after one year was not associated with later recovery from probable PTSD. To the best of our knowledge, this is the first study to examine the association between BMI and probable PTSD recovery.

Although no previous studies have examined the association between BMI and PTSD recovery, systematic reviews and meta-analyses have suggested the effect of PTSD on obesity and the risk of weight gain^5,11,18^. While the specific mechanisms remain unknown, previous studies have reported common risk factors between obesity and PTSD. Consumption of healthy diets, including fish (omega-3 polyunsaturated fatty acids), meat, and vegetable, is negatively associated with both obesity and PTSD^5–8^. PTSD is associated with low-density lipoprotein-cholesterol, cortisol, interleukin (IL)-2, IL-6, IL-8, leptin, insulin resistance, and tumor necrosis factor (TNF)-α^9–12^. PTSD is caused by neuroendocrine links, leading to the activation of the sympathetic-adrenergic nervous system together with the release of hormones via the endocrine hypothalamic–pituitary–adrenal (HPA) axis^12,19^. A previous study also showed HPA axis dysregulation, with decreased blood and urinary cortisol levels and enhanced HPA axis sensitivity to negative feedback^12,20^. Lipid metabolism is also affected by these alterations^21^. Additionally, microglial TNF-α was associated with sustained fear memory, which is a cause of PTSD^22^. A process of fear memory formation changes the proinflammatory cytokine production in the brain. It was observed that TNF-α increased in mice which retained fear memory, whereas it returned to basal levels in mice, which extinguished fear memory. Previous studies showed that pharmacological treatments that target inflammatory mechanisms are associated with decreased for having a diagnosis of PTSD and lower levels of PTSD symptoms in traumatized participants^12,23^. Interventions to improve obesity might also prevent from new onset and prolonged PTSD because obesity, especially the accumulation of visceral fat, increases the levels of the abovementioned metabolic substances^8,12^. Moreover, a certain personality is associated with both obesity and PTSD^13–17^. Odds Ratio and 95% CIs of PTSD increased in individuals with nervousness (OR 1.09, 95% CI 1.01–1.17)^13^. A systematic review reported neuroticism as a risk factor for obesity^16^. Therefore, obese participants would find it difficult to recover from PTSD owing to the above factors mediating obesity and PTSD.

Meanwhile, obesity in participants who did not recover from probable PTSD during 2012–2013 was not associated with recovery in 2014. BMI and the prevalence of obesity increased dramatically in victims after the disaster^1^. However, the victims received support for the recovery of their quality of health. The Fukushima Health Management Survey has conducted health checkups^24^, consultation meetings, and lecture presentations and has distributed informational health brochures. Not infrequently victims have decreased BMI and improved obesity owing to these interventions. Therefore, misclassification of BMI changes in the follow-up period might induce attenuation of the association between BMI and probable PTSD in these participants.

Our study had several limitations. First, information on probable PTSD before the disaster was not available. Some study participants might have had probable PTSD not associated with the disaster. Second, probable PTSD were assessed almost one year after the disaster. Thus, participants who developed and recovered from probable PTSD before this assessment were excluded. Therefore, this study included study participants limited by severe probable PTSD persisting for a long period after excluding potential participants with relatively mild probable PTSD. Third, the survey periods differed between the assessments of BMI and probable PTSD. Probable PTSD was assessed using the Mental Health and Lifestyle Survey from January to May in 2012, while BMI was measured as part of the Comprehensive Health Check from July 2011 to March 2012. Thus, the results might show weak associations because of underestimation owing to non-differential misclassification. The survey period did not depend on exposure and outcome. Fourth, respondents of the Mental Health and Lifestyle Survey comprised 41.2% of the target population. Therefore, the results of this study may be affected by a response bias. Individuals with PTSD were more likely to not respond. However, this response tendency was not associated with BMI; therefore, while the present results underestimated the absolute risk, this bias did not affect the relative risk. Fifth, the number of participants in the underweight and obesity is relatively small compared with that in the normal weight and overweight. The accuracy of the results might be unstable. Further larger studies are needs to clarify the association between BMI and recovery from probable PTSD.

In conclusion, the results of this study showed that obesity may be useful as a predictor for probable PTSD recovery. After disasters, both physical and mental health are important public health concerns for victims. Therefore, considering BMI might be effective to improve mental health. The obese victims with PTSD might be required more intensive support and careful follow-up to recover it. Weight control has a possibility of benefit for PTSD.

## Methods

### Study participants

**Figure 1:**
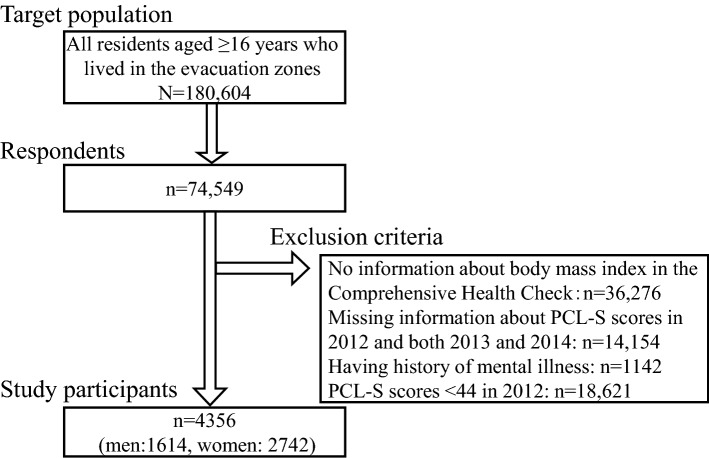
Flow diagram of the study participants.

This study merged data from the Mental Health and Lifestyle Survey and Comprehensive Health Check in the Fukushima Health Management Survey. The details of the Fukushima Health Management Survey have been described elsewhere^24^. Briefly, the Mental Health and Lifestyle Survey was a self-administered questionnaire that annually assessed mental health and various lifestyle habits according to age category (0–6, 7–15, and ≥ 16 years); the first survey was delivered in January 2012 to all residents who lived in the evacuation zones owing to the radiation accident in Fukushima Prefecture on March 11, 2011. The evacuation zone was a government-designated area with a radius of 20 km around the nuclear power plant. PCL-S scores did not assess in participants aged 0–6 and 7–15 years. In those aged ≥ 16 years, PCL-S scores were not assessed after 2015. Among the target population of 180,604 individuals aged ≥ 16 years in 2012, 74,549 (41.2%) responded. The study participants included 38,273 respondents for whom information about body mass index (BMI) was available from the Comprehensive Health Check. The present analysis excluded participants with missing PCL-S data in 2012 and both 2013 and 2014 (n = 14,154), with a history of mental illness (n = 1142), and with PCL-S scores < 44 in 2012 (n = 18,621). Thus, we analyzed a total of 4356 participants (1614 men and 2742 women) (Fig. 1).

### Ethical issue

The study protocol was approved by the Ethics Committee of Fukushima Medical University (29064). The questionnaires were described, and an explanation of the survey and handling of personal information was provided on a cover page. The participants subsequently provided their written informed consent to participate by returning the questionnaires. In participants aged < 18 years, their parents or representative also provided written informed consent. This study was conducted according to the Declaration of Helsinki. We followed the STROBE Statement to report our observational study.

### BMI

BMI was calculated as the measured weight divided by the square of measured height. We divided the participants according to the following BMI categories: < 18.5 kg/m^2^ (underweight), 18.5–24.9 kg/m^2^ (normal weight), 25.0–29.9 kg/m^2^ (overweight), and ≥ 30.0 kg/m^2^ (obese)^25^.

### Probable PTSD

Probable PTSD were defined by PCL scores, which is used for screening PTSD, aiding in the diagnostic assessment of PTSD, and monitoring changes in probable PTSD. Among the three versions of PCL in the Diagnostic and Statistical Manual of Mental Disorders, Fourth Edition, the present survey used PCL-S^4,26^.

Probable PTSD were defined as PCL-S scores ≥ 44; the scores are derived from a 17-item self-report measure according to the recommendation for diagnostic efficiency^2,4,26,27^. Participants responded to each item as follows: 1 (not at all), 2 (slightly), 3 (moderately), 4 (quite a lot), or 5 (very much) in the past month. Therefore, PCL-S scores < 44 were used to denote recovery from probable PTSD during the follow-up period.

### Statistical analysis

We used Poisson regression with robust error variance to derive prevalence ratios (PRs) and 95% confidence intervals (CIs) for recovery from probable PTSD (PCL-S scores < 44) in 2013 and 2014, respectively, according to BMI categories and to adjust for potential confounding factors. This analysis was performed using SAS version 9.4^28^. The normal weight category was selected as the reference. The p-values for trends were calculated for continuous variables. All p-values were two-tailed, and *p* < 0.05 indicated statistically significant differences.

We considered the following variables as potential confounding factors: sex, age (< 30, 30–39, 40–49, 50–59, 60–69, or ≥ 70 years), PCL-S scores in 2012 (continuous), smoking (never smoked, quit, or current smoker), drinking (don't drink or only rarely, quit, or drink at least once a month), exercise habit (almost every day, 2–4 times/week, once/week, or almost never), sleeping habit (satisfied, slightly dissatisfied, quite dissatisfied, or very dissatisfied or haven't slept at all), education level (elementary school・Junior high school, high school, vocational college/junior college, or university・graduate school), activities of daily living (independent or dependent), history of cancer or heart disease (yes or not), house damage (no damage, partial damage or partial collapse, or partial but extensive collapse or total collapse), lose someone close in disaster (yes or not), experience about tsunami following the GEJE (yes or no), and experience about nuclear reactor accident (heard the explosion) following the GEJE (yes or not). Multiple imputation using 20 iterations was applied for missing covariate information.

Additionally, we repeated the above analysis to examine the association between BMI and recovery of probable PTSD in 2014 among participants who had probable PTSD until 2013.

This analysis was also conducted after excluding participants aged < 20 years because the smoking and drinking statuses of all participants in this age group should be categorized as “never” and “almost never.”

## Supplementary Information

Supplementary Information

## Abbreviations

BMI: Body mass index
PTSD: Post-traumatic stress disorder
PCL-S: Post-traumatic stress disorder checklists-specific
PRs: Prevalence ratios
CIs: Confidence intervals
SD: Standard deviation
GEJE: The great east japan earthquake
IL: Interleukin
TNF: Tumor necrosis factor
HPA: Hypothalamic-pituitary-adrenal
ORs: Odds ratios
