# Supplementary material for: The association between body mass index and recovery from post-traumatic stress disorder after the nuclear accident in Fukushima
Source: Sci Rep. 2021 Mar 5;11:5330. doi: 10.1038/s41598-021-84644-5 (PMC7935866; doi:10.1038/s41598-021-84644-5)
Supplement: Supplementary file 1 — Supplementary Information [file 41598_2021_84644_MOESM1_ESM.docx]

**The Association between Body Mass Index and Recovery from Post-traumatic Stress Disorder after the Nuclear Accident in Fukushima**

Running Title: BMI and recovery from PTSD

Masato Nagai, PhD^1, 2^, Tetsuya Ohira, MD, PhD^2, 3^, Masaharu Maeda, MD, PhD^3, 4^, Seiji Yasumura, MD, PhD^5^, Itaru Miura, MD, PhD^6^, Shuntaro Itagaki, MD, PhD^6^, Mayumi Harigane, PhD^3, 5^, Kanae Takase, PhD^3, 7^, Hirooki Yabe, MD, PhD^7^, Akira Sakai, MD, PhD^3, 8^, Kenji Kamiya, MD, PhD^3, 9^

^1^ Department of International and Community Oral Health, Tohoku University Graduate School of Dentistry, Sendai, Japan

^2^ Department of Epidemiology, Fukushima Medical University School of Medicine, Fukushima, Japan

^3^ Radiation Medical Science Center for the Fukushima Health Management Survey, Fukushima Medical University, Fukushima, Japan

^4^ Department of Disaster Psychiatry, Fukushima Medical University School of Medicine, Fukushima, Japan

^5^ Department of Public Health, Fukushima Medical University School of Medicine, Fukushima, Japan

^6^ Department of Neuropsychiatry, Fukushima Medical University School of Medicine, Fukushima, Japan

^7^ Department of Public Health and Home Care Nursing, Fukushima Medical University, Japan

^8^ Department of Radiation Life Science, Fukushima Medical University School of Medicine, Fukushima, Japan

^9^ Hiroshima University, Higashihiroshima, Japan

Address for correspondence:

Masato Nagai, [PhD.](javascript:goWordLink(%22M.Ed%22))

Department of International and Community Oral Health, Tohoku University Graduate School of Dentistry, Sendai, Japan

4-1, Seiryo-machi, Aoba-ku, Sendai, Miyagi 980-8575, Japan

Phone: +81-22-717-7639

Fax: +81-22-717-7644

E-mail: [m-nagai@med.tohoku.ac.jp](mailto:m-nagai@med.tohoku.ac.jp)

Manuscript information:

#Financial support: This survey was (partly) supported by the National Health Fund for Children and Adults Affected by the Nuclear Incident, and the Program of the Network-type Joint Usage/Research Center for Radiation Disaster Medical Science.

#Word count: 2,072

#References: 28

#Tables: 3

#Figures: 1

#Supplemental tables: 2

**Abbreviations list**

BMI: body mass index, PTSD: post-traumatic stress disorder, PCL-S: Post-traumatic Stress Disorder Checklists-specific, PRs: prevalence ratios, CIs: confidence intervals, SD: standard deviation, IL: interleukin, TNF: tumor necrosis factor, HPA: hypothalamic-pituitary-adrenal, ORs: odds ratios
